# The Growing Burden of Informal Caregivers During COVID-19

**DOI:** 10.1093/geroni/igab046.3446

**Published:** 2021-12-17

**Authors:** Stephanie MacLeod

**Affiliations:** UnitedHealth Group, United States

## Abstract

Caregiver burden has negative effects on health outcomes and quality of life. Meanwhile, safety protocols during the COVID-19 pandemic created immediate impacts on informal caregiving with increasing burden on family caregivers. Our primary purpose was to describe the impacts of the pandemic on caregiver burden among informal caregivers, and their sudden shift in roles as a result. This review describes emerging effects on various aspects of health and explores future directions to support informal caregivers. A streamlined search was conducted to fit the scope of this review, with key terms determined to identify relevant publications. Common research databases and mainstream resources were utilized. We focused on research published since March 2020 to align with the timing of the pandemic in the US. Early research suggests that the pandemic has worsened caregiver burden among informal family caregivers. Reported health impacts include greater stress, pain, depression, sleep problems, and irritability, decreased social connectedness and quality of life. Informal family caregivers face negative health outcomes and distress as a result of greater caregiver burden and intensity during the COVID-19 pandemic. Immediate solutions are needed to alleviate this growing burden and provide ongoing support. Future work should explore the potential of boosting positive resources such as resilience and purpose to ease caregiver burden.

